# Relations between risk perception, perceptions of peers’ driving, and risky driving among Cambodian adolescents

**DOI:** 10.3389/fpsyg.2023.1238945

**Published:** 2023-08-16

**Authors:** Bouyheak Lim, Cindy J. Lahar, Hoang-Minh Dang, Bahr Weiss

**Affiliations:** ^1^Child and Adolescent Clinical Psychology Program, VNU University of Education, Hanoi, Vietnam; ^2^Psychology Program, University of South Carolina Beaufort, Bluffton, SC, United States; ^3^Clinical Research Institute for Society, Psychology and Education, VNU University of Education, Hanoi, Vietnam; ^4^Department of Psychology and Human Development, Vanderbilt University, Nashville, TN, United States

**Keywords:** risky driving, risk perception, perceptions of peers’ driving, Cambodia, adolescents, social norms

## Abstract

**Introduction:**

Traffic accidents are a leading cause of death globally, with substantial economic impact particularly in low-and-middle-income countries (LMIC). Adolescents are at particular risk, partly due to their tendency to engage in risky driving. However, most research designed to identify potential causes of risky adolescent driving has been conducted in Western, high-income countries, which often have substantial cultural differences from LMIC that potentially influence risky adolescent driving.

**Methods:**

The present study, one of the first focused on this topic in Southeast Asia, cross-sectionally assessed 425 adolescent motorbike drivers in the Southeast Asian LMIC Cambodia. Adolescents’ (a) beliefs about peers’ driving (social norms) and (b) driving risk perception were assessed as predictors of four risky driving behaviors: aggressive driving; distracted driving; intoxicated driving; violating driving laws.

**Results:**

Canonical correlation analysis identified a general relation between (a) beliefs about peers’ driving, and (b) all four risky driving behaviors, with *R*^2^ = 0.35 indicating over one-third of the variance in risky driving was explained by perceptions of peers’ driving. Risk perception was not involved in the significant canonical relation, however. Gender moderated two of the underlying relations, with females showing larger relations between perceptions of friends’ driving, and distracted driving and violating driving laws.

**Discussion:**

These findings provide useful directions for future research (e.g., assessing the accuracy of Cambodian adolescents’ perceptions of peers’ driving) useful for helping stakeholders tailor road safety programs (e.g., providing adolescent drivers with accurate information regarding their peers’ actual driving behaviors) for adolescent motorcyclists in Cambodia and similar countries.

## Introduction

Traffic accidents are a major global concern, the eighth leading overall cause of death worldwide and the leading cause of death among youth (World Health Organization, 2015, 2018). Risk of traffic fatalities is significantly higher in low-and middle-income countries (LMIC) and it has been estimated that in LMIC traffic accidents result in a loss of approximately 5% of their GDP (World Health Organization, 2015, 2018). The majority of traffic accidents are among young people (aged 15–29), which increases the economic effects of traffic accidents since economic productivity is often highest earlier in life, and the long-term effects of disability or injury are more substantial earlier in life (World Health Organization, 2018).

Road traffic accidents are primarily caused by human behavior and attitudes, rather than by equipment failure, etc. (World Health Organization, 2018). “Risky driving behavior” refers to actions that a driver performs that are related to an increased risk of a road accident resulting in potential injury or fatality for the driver or other persons (Schmidt, 2012).

As noted above, young people are at particular risk for road traffic accidents (World Health Organization, 2018). One factor related to youth traffic safety risk is perceptions of peers’ driving behavior, perceived social norms for risky driving. Youth in general are strongly influenced by their peers’ behavior, via processes such as behavioral modeling, etc., including in relation to road safety behaviors. For instance, adolescents have been found to be more likely to engage in distracted driving behaviors if they believe that their peers engage in and approve of similar behaviors (Carter et al., 2014). Simons-Morton et al. (2019) conducted a simulating driving study among U.S. adolescents. In this study, peer norms related to risky driving were experimentally manipulated through use of a confederate “passenger” in a simulated driving task who either supported risky driving (positive social norms toward risky driving) or presented as risk averse (negative social norms toward risky driving). Results indicated that the adolescent “driver” was more likely to engage in risky driving behavior (e.g., speeding pass a slow vehicle) when the “passenger” presented positive social norms for risky driving. Thus, driver perceptions regarding their peers’ risky driving behaviors and peers’ attitudes toward such behaviors are a factor important for understanding youth risky driving.

Another important predictor of risky driving behavior is “risk perception,” which refers to how individuals perceive the likelihood of occurrence and severity of negative consequences of a behavior (Ferrer and Klein, 2015). Risk perception has been found in a number of studies to be a strong predictor of risky driving behaviors (e.g., Carter et al., 2014). For instance, Duarte and Mouro (2019) found a negative correlation among Portuguese drivers between the perceived risk of a driving behavior (e.g., speeding; driving when tired) and the likelihood of engaging in the behavior; i.e., the higher the risk perception for the driving behavior, the lower the likelihood that the driver would engage in the behavior. Harbeck and Glendon (2018) similarly found a strong negative correlation between risk perception and engagement in risky driving behaviors. Thus, risk perception also appears to be a key factor in risky driving.

Although the majority of serious traffic injuries and fatalities occur in LMIC, and although the consequences of traffic accidents are most substantial in LMIC, the majority of research investigating human behavioral causes of traffic accidents has been conducted in high-income countries (HIC) such as Australia, European countries, and North America (World Health Organization, 2018). This represents a significant limitation as generalizability of research results between Western HIC and non-Western LMIC is unclear, given that social factors (e.g., social norms, perceptions of peer behaviors, risk perception) can be influenced by culture (Haghdoust et al., 2022). The present study is one of the first investigations in this area to be conducted in Southeast Asia, a region with over 688 million inhabitants (Worldometer, 2023). The study examined relations between risky driving behaviors, and drivers’ risk perception and beliefs about peers’ driving behaviors, among adolescents in Cambodia, a Southeast Asian LMIC. Traffic accidents have been recognized as a major public health concern in Cambodia (Dy, 2016; United Nations Development Program – Cambodia, 2021). In Cambodia, which has a population of approximately 17 million people, during the first 6-months in 2018 police stopped 102,995 vehicles for potential safety violations (World Health Organization, 2018). Most recently, in the first half of 2021 over 700 traffic fatalities were reported in over 1200 road accidents across Cambodia, with risky driving behaviors (e.g., speeding; careless driving; ignoring the right of way) responsible for the large majority of the accidents (Kimmarita, 2021).

Another limitation in this literature is that much of the research has focused on automobiles. Although automobile driving is clearly an important component of traffic safety, motorbike accidents are a particular public health concern due to the greater risk for the driver and passengers associated with motorbike as compared to automobile accidents; (i.e., the lack of protection provided by the body of the car for motorbike drivers and passengers; World Health Organization, 2018). In Cambodia, during the first 6-months of 2018 police stopped 77,795 motorbike drivers for violating driving safety laws. The present study focused on motorbike drivers, because of the high rates of motorbike road accidents throughout Cambodia and other LMIC (Dy, 2016; United Nations Development Program – Cambodia, 2021). The study focused on adolescents at least 16 years of age, which is the legal minimum for driving a motorbike, because of the increased risk of serious vehicle accidents among youth (World Health Organization, 2018).

Based on the literature review, the following hypotheses were made for the present study: (a) All four risky driving behaviors would load on one canonical variate; (b) Beliefs about Peers’ Driving and Risk Perception would load on separate canonical variates; (c) the canonical correlation involving Beliefs about Peers’ Driving would be significant; (d) the canonical correlation involving Risk Perception would be non-significant, given mixed evidence in the literature for effects of risk perception of driving; and (e) several of the Gender moderator effects would be significant, but given the complexity of the literature and the close links between Gender and culture (e.g., van de Water et al., 2016), more specific hypotheses regarding Gender were not made.

## Materials and methods

### Sampling frame and participants

The purpose of the sampling frame was to obtain a sample of Cambodian adolescent drivers reflective of the diversity of Phnom Penh, the capital city of Cambodia. Sample inclusion criteria were structured to provide participants familiar with driving a motorbike, and included participants that (a) drive a motorbike to school at least 4 days per week, and (b) had been driving a motorbike for at least 6 months. Students in Grade 11 were targeted, because in Grade 12 students in Cambodia are preparing for college entrance exams and generally do not have time for non-academic activities. Four hundred twenty-five adolescents ages 16 to 18 were surveyed at four high schools purposively selected to be distributed geographically across Phnom Penh. Fifty percent of the adolescents were female, with a mean age of the sample of 16.95 (*SD* = 0.68) years. On average, the adolescents had been driving for 2.70 years (*SD* = 1.77), with most adolescents (81%) reporting driving a motorbike for between 1 and 4 years.

### Assessment instruments

The study survey included measures assessing background demographics, risky driving behaviors (as the dependent variables), and driving behavior risk perception and beliefs about peers’ driving behaviors (as the independent variables). With the exception of the demographics measure, the questionnaires had been developed in English. When social science measures are used cross-culturally, in order to ensure that the instrument accurately reflects what it is intended to measure, it is necessary that the measure be culturally adapted (Gjersing et al., 2010). Consequently, measures in the present study were culturally adapted and translated into Khmer using standard procedures to maintain the measures’ semantic, technical, and conceptual content (Hambleton et al., 2005; Byrne, 2016). Adaptation involved four phases. First, measures were adapted for Cambodia by a team of U.S. and Cambodian psychologists, with the goal of ensuring the cultural appropriateness of the measures. For instance, questions of automobile risky driving were adapted for the Cambodian context of motorcycle driving. Second, measures were translated from English into Khmer by the first author, a native Khmer speaker fluent in English, with a master’s degree in clinical psychology obtained in an English language program. The research team collaborated in this process to clarify any unclear wording in the original English measures. Third, measures were independently back-translated into English by two native Khmer-speaking psychologists also with an English language master’s degree in psychology, both of whom with several years of experience in conducting similar translations. Finally, the penultimate Khmer versions were piloted with 37 Cambodian undergraduate students at the Royal University of Phnom Penh, who were asked to identify any confusing or misleading wording, which were accordingly modified.

#### Risky driving behaviors

The *Risky Driving Behaviors* (RDB) questionnaire used in the present study was adapted from Schmidt’s *Youth Domains of Risky Driving Scale* (Schmidt, 2012). The YDRSD is a 58-item self-report measure using a five-point Likert scale, ranging from 1 (never) to 5 (very often), that measures four domains of risky driving behaviors (aggressive driving; distracted driving; substance use; violating driving laws). The scale was adapted for the present study in order that it focus on the context of youth driving in Cambodia. For instance, items from the YDRSD focusing on risky automobile driving (e.g., driving without wearing a seatbelt) were dropped. Several items specific to the Cambodian context not included in the YDRSD were added (e.g., driving a motorbike the wrong way down a one way street), resulting in 31 items. The *Aggressive Driving* subscale has eight items, such as “… yelled at another driver in anger or frustration.” The *Distracted Driving* subscale has nine items, such as “… talked to passengers while driving.” The *Intoxicated Driving* subscale has four items, such as “… driven after consuming more than one alcoholic beverage.” The *Violating the Law Driving* subscale has ten items, such as “…changed lanes or turned without signaling.” Respondents rated each item on a five-point Likert scale for how frequently they had engaged in each behavior over the past 6 months. Chronbach’s internal consistency alpha reliability was 0.89 for the total score, and for the subscales ranged from 0.72 to 0.76.

#### Risk perception

Risk perception has been defined as an individual’s perception of risk of negative outcomes associated with a particular action or event (Ferrer and Klein, 2015). In this study, risk perception was assessed as the degree of danger perceived by the participant regarding various risky driving behaviors (e.g., going through a red light). Items for the current study’s risk perception questionnaire were adapted from the 10-item risk perception scale developed by Ivers et al. (2009), and from the 13-item risk perception scale developed by Simons-Morton et al. (2014). The present study’s measure included 11 items, with responses rated by the participant on a Likert scale rating from 0 (not dangerous) to 3 (very dangerous). For example, one item asked the respondents to report how dangerous they thought it is to drive while talking on a cellphone. The Chronbach’s alpha internal reliability coefficient for the risk perception scale in this study was α = 0.92.

#### Beliefs about peer’s driving and attitudes

Beliefs about peers’ risky driving and their attitudes toward such behaviors were measured using the 13-item *Perceptions of Peers’ Driving* scale. The scale was based on the 14-item *Risky Driving Behaviors* scale by Ivers et al. (2009), with several items deleted, edited, or added to fit with the Cambodian context. Items include “How often do your friends drive without wearing a helmet” and “How often do your friends drive fast just for fun.” Respondents rate each item on a five-point Likert scale ranging from 0 (never) to 4 (very often). Chronbach’s alpha internal reliability in the present sample was α = 0.87.

### Procedures

The present study was reviewed and approved by the Cambodian National Ethics Research Committee. Purposive sampling was used to select four high schools geographically distributed across Phnom Penh, Cambodia. All four schools identified agreed to participate in the study. After receiving school permission, Grade 11 classroom teachers in the schools were contacted and informed about the study. All teachers contacted agreed for their classroom to participate, and distributed a detailed study description and parental consent forms to students interested in potentially participating. The study description included the study selection criteria for the students, that the student: (a) drive a motorbike to school at least 4 days per week, and (b) had been driving a motorbike for at least 6 months. Students with parental consent and who themselves provided assent completed the study questionnaire, which was in Khmer, with the researcher present in the classroom to provide clarification for any items, during a free period. The participants spent about 30 to 60 min completing the survey. Participants received a small gift such as a pen or highlighter for their participation in the study.

## Results

### Descriptive analyses

All analyses were conducted in SAS 9.4. Bivariate correlations between *Age* and *Gender*, and *Risk Perception*, *Perceptions of Peers’ Driving*, and the *Risky Driving Behaviors* are reported in Table 1. *Gender* was coded as female = 1, male = 2; thus a positive correlation indicates higher levels of the construct reported by males. The only driving-related variable significantly correlated with *Age* was *Intoxicated Driving.* Males reported higher levels *Peers’ Risky Driving, Intoxicated Driving*, and *Violating the Law Driving* (i.e., there was a positive correlation), and females reported higher levels of *Risk Perception. Perceptions of Peers’ Driving* behaviors were significantly correlated with all four Risky Driving Behaviors (see Table 1). Correlations ranged from 0.32 (for *Perceptions of Peers’ Driving* and *Intoxicated Driving*) to 0.51 (for *Perceptions of Peers’ Driving* and *Violating the Law Driving*). All of these correlations were positive, indicating that the more an adolescent believed that peers engaged in and approved of risky driving, the higher the level of risky driving behaviors in which the participant reported themselves engaging. *Risk Perception* was not significantly correlated with any *Risky Driving Behavior*; i.e., the extent to which an adolescent reported that risky driving behaviors were dangerous was unrelated to their scores on any risky driving behaviors.

**TABLE 1:** Bivariate pearson correlation coefficients (*N* = 425).

	Age	Gender^1^	Peers’ risky driving	Risk perception	Aggressive driving	Distracted driving	Intoxicated driving	Violating driving laws
Age	1.00							
Gender	0.13**	1.00						
Peers’ risky driving	0.00	0.27****	1.00					
Risk perception	-0.05	-0.16***	0.09	1.00				
Aggressive driving	0.01	0.09	0.51****	-0.06	1.00			
Distracted driving	0.08	0.07	0.44****	-0.03	0.56****	1.00		
Intoxicated driving	0.10*	0.15**	0.35****	-0.07	0.36****	0.41****	1.00	
Violating driving laws	-0.02	0.15**	0.51****	-0.06	0.66****	0.63****	0.38****	1.00

^1^For gender, female = 1 and male = 2; i.e., a positive correlation indicates higher scores for males. *p < 0.05; **p < 0.01; ***p < 0.001; ****p < 0.0001.

### Primary analyses

#### Canonical correlation analyses

The primary analyses summarized the bivariate relations, using a canonical correlation analysis conducted with SAS Proc Cancorr, with a 0.40 loading cutoff. Canonical correlation analysis identifies relations between two sets of variables, in the present case, (*Set 1*) *Risk Perception* and *Perceptions of Peers’ Driving*, and (*Set 2*) the four risky driving behaviors. Canonical correlation analysis extracts linear combinations within each set of variables that are maximally correlated with each other across the two sets of variables (Olive, 2017). The loadings of each variable on its canonical variate represent, as in factor analysis, the relation (often presented as a correlation) between the item and the canonical variate. In the present analyses, the first canonical relation was significant, with *F*_(8,838)_ = 25.34, *p* < 0.0001, *R* = 0.59. As Table 2 indicates, this effect reflected a relation between *Perceptions of Peers’ Driving* (with a loading of 0.98) and all four of the Risky Driving Behaviors (with loadings ranging from 0.62 to 0.88).; *Risk Perception* did not load significantly on this dimension, with a loading of −0.12. The second canonical relation was non-significant, *F*_(3,420)_ = 0.25. Thus, there was a strong relation between perceptions of peers’ driving and risky driving, but not between risk perception and risky driving. In order to test whether these relations might be due at least in part to confounding between the geographic location within Phnom Penh and driving behaviors, these analyses also were conducted including High School as a covariate. Because canonical correlations require continuous variables, High School was converted to three 1 degree of freedom contrasts. However, inclusion of High School resulted in no changes in the significance of any of the inferential tests in the analyses, and parameter estimates showed minimal changes; i.e., High School had essentially no effect on analyses.

**TABLE 2:** Canonical correlation analyses.

	Canonical variate	
	#1	#2
Canonical correlation	0.59****	0.04
**Risk factors**		
Perceptions of peers’ driving	**0.98**	0.20
Risk perception	-0.12	**0.99**
**Risky driving behaviors**		
Aggressive driving	**0.88**	0.05
Distracted driving	**0.76**	**0.46**
Intoxicated driving	**0.62**	**–0.61**
Violating driving laws	**0.88**	0.12

****p < 0.0001 for canonical correlations. Canonical coefficients are in the metric of a correlation. Bolded coefficients are above the 0.40 loading cutoff.

#### Gender as moderator

As noted in the Introduction (above), gender has been found to influence risky driving behavior (e.g., Latif et al., 2017). In order to determine the extent to which gender influenced relations in the present study, gender was assessed as a moderator of the effects of peers’ driving and risk perception on the risky driving behaviors, using general linear model analyses. Two moderator effects were significant. The *Perceptions of Peers’ Driving* X *Gender* interaction was significant for *Distracted Driving*, *F*_(1,418)_ = 8.82, *p* < 0.005. The *Perceptions of Peers’ Driving* X *Gender* interaction also was significant for *Violating the Law Driving*, *F*_(1,418)_ = 7.34, *p* < 0.01. The other interactions were non-significant. To interpret the significant interactions, separate regression analyses between the independent variable (*Perceptions of Peers’ Driving*) and the dependent variables (*Distracted Driving*; *Violating the Law Driving*) were conducted for females and males. For *Distracted Driving*, the effect of *Perceptions of Peers’ Driving* was significant both for females, *F*_(1,207)_ = 78.73, *p* < 0.0001, β = 0.52 and for males, *F*_(1,211)_ = 36.35, *p* < 0.0001, β = 0.38. Thus, although the relation between *Perceptions of Peers’ Driving* and *Distracted Driving* was significant for both genders, the effect was larger for females. Similarly, for *Violating the Law Driving*, the effect of *Perceptions of Peers’ Driving* was significant both for females, *F*_(1,207)_ = 113.25, *p* < 0.0001, β = 0.59, and for males, *F*_(1,211)_ = 44.86, *p* < 0.0001, β = 0.42, with the effect larger for females. As with the canonical correlation analyses, in order to test whether any these results might have been influenced by confounding between with the geographic location within Phnom Penh, these analyses also were conducted including High School as a covariate. In this case, because these were general linear model analyses, High School was included as a 3 degree of freedom categorical variable. As with the canonical correlation analyses, inclusion of High School resulted in no changes in the significance of any of the inferential tests in the analyses, and parameter estimates again were minimally changed; i.e., including High School had essentially no effect on the analyses.

## Discussion

In this study, the canonical correlation analysis produced a significant canonical relation between Cambodian adolescents’ (a) perceptions (social norms) of their peers’ driving and (b) their own risky driving behaviors. The *R*^2^ statistic (0.35) indicated that over one-third of the variance in the adolescents’ self-reported risky driving (*Aggressive Driving*, *Distracted Driving*, *Intoxicated Driving*, *Violating the Law Driving*) can be statistically explained by their perceptions of their peers’ driving, which is a substantial relation. An important aspect of this relation is that it involved all of the risky driving behaviors, with strong loadings (0.62 to 0.88) on the canonical variate. This is similar to some previous findings in this area (e.g., Carter et al., 2014). This result provides for important potential intervention targets. Research has found that perceptions of social norms regarding peers’ maladaptive and/or anti-social behavior—including risky driving—often over-estimate the frequency of the peers’ behavior. Geber et al. (2021), for instance, found that young German drivers significantly over-estimated the frequency with which their peers engaged in risky driving behaviors (e.g., texting while driving; speeding), in some cases by more than 350% (for drinking and driving). This supports development of social norms interventions (Berkowtiz, 2004) to reduce risky driving. Research in Cambodia similar to Geber et al. (2021) to identify local discrepancies between perceived and actual social norms will be an important next step in Cambodia and other similar countries for development of interventions to reduce risky driving among adolescents.

In contrast to perceived social norms, adolescents’ perceptions of the danger associated with risky driving were not significantly related to any of the four categories of risky driving behaviors. Although in general prior research has found negative relations between risk perception and risky driving (e.g., Carter et al., 2014; Harbeck and Glendon, 2018; Duarte and Mouro, 2019), the literature is not fully consistent, and in some cases prior research has found correlations that have been positive, or not significantly different from zero (e.g., O’Brien and Gormley, 2016; Song et al., 2021), as is the case in the present study. There are several possible reasons for the variability of results across studies. First, it is possible or even likely that “risk” is a multi-dimensional construct, including components such as the (a) likelihood of occurrence and (b) severity of consequences (Cordellieri et al., 2016). These components may have different effects on risky driving. For example, perceptions of a high likelihood of occurrence but low severity of consequences might result in minimal relations between “risk perception” and risky driving (Cordellieri et al., 2016). In contrast, a high likelihood of occurrence but high severity of consequences might result in strong negative relations between risk perception and risky driving (Cordellieri et al., 2016). A second possible factor underlying variability in the relation between risk perception and risky driving may be the perceived controllability of the consequences of risky driving. Cheng and Ng (2012), for example, found that among Hong Kong motorbike drivers, one factor influencing risky driving was the extent to which traffic accidents were perceived as avoidable. They found that many motorbike drivers attributed causes of driving accidents to uncontrollable factors (often “mystical” or “superstitious” beliefs), ultimately reducing the relation between risk perception and risky driving behavior (Cheng and Ng, 2012).

These possibilities suggest important areas for future research to reduce risky driving among adolescents in Cambodia, and similar countries. Such research should include assessing multiple components of “risk,” including probability of occurrence, severity of consequences, and controllability (Cordellieri et al., 2016), and determining their relations to risky driving. Using this information, preliminary intervention programs that highlight the concrete consequences of accidents for both the driver as well as others may be useful, but should be accompanied by solution-focused, action-oriented, and self-confidence-building measures so that the outcome-focused messages do not lead to reactance (i.e., increased risky driving) (von Beesten and Bresges, 2022). Development of such interventions is complex, and must fit with the local culture, providing factual information without generating negative reactance to the message (Dahlgren, 2022).

The study also found that some underlying components of the canonical relation between perceptions of peers’ driving behaviors and risky driving differed as a function of gender. Specifically, although females and males both showed significant relations, females showed statistically larger relations between (a) *Perceptions of Peers’ Driving*, and (b) *Distracted Driving* and *Violating the Law Driving*. This may reflect the fact that in general, females are in some circumstances more influenced by social relationships than males (Tulviste et al., 2010). However, the extent to which these peer influences impact gender effects on risky driving is complex. In Simons-Morton et al. (2019) simulating driving study, there were several significant gender differences in the relation between risky driving and the experimentally manipulated peer norms for risky driving. Compared to males in the “risk averse passenger” condition (low social norms for risky driving), males in the “risk accepting passenger” condition (high social norms for risky driving) were more likely to not stop at a red light and to speed past a slowly moving vehicle. Females showed a similar but smaller effect for passing a slow vehicle, but not for failing to stop at a red light.

More generally, gender effects on risky driving and moderator effects have been complex and not highly consistent across studies and countries (Cordellieri et al., 2016). For instance, in a U.S. adolescent sample, Rhodes and Pivik (2011) found that the relation between risk perception and risky driving was moderated by Gender, with females showing significantly stronger effects of risk perception on risky driving. Similarly, Cordellieri et al. (2016) found in a sample of youth from nine European countries that females’ risky driving attitudes were more influenced by their risk perception than males’ were. However, in the present study, no Gender moderator effects on the effects of risk perception on risky driving were significant. The reasons for the differences between the present study, and these and other similar studies are likely complex, given how closely linked gender roles and behaviors are to culture (van de Water et al., 2016). Thus, cross-cultural research focused on identifying the effects of culture the effects of gender con risky driving, in particular its effects as a moderator, will be important.

A related complexity within the present study’s results is why effects of perceived social norms for risky driving on *Distracted Driving* and *Violating Driving Laws* differed by gender but not for *Aggressive Driving*, and *Intoxicated Driving*. One possible explanation may be that the latter two (driving) behaviors are more closely connected to broader personality constructs related to anti-social personality traits (American Psychiatric Association, 2013). That is, alcohol drinking among adolescents and aggressive behavior may be more closely linked to broader, anti-social personality constructs non-specific to driving, hence reducing gender-related peer effects. Thus, one important area for future research will be to assess the effects of key personality and cultural constructs that may influence or moderate the effects of the psychosocial risk factors on risky driving. For instance, in a sample of Italian adolescents Lucidi et al. (2019) found that various personality characteristics (e.g., altruism, normlessness) had significant effects on risky driving behaviors and attitudes. Understanding how such factors moderate risk factors and link to gender will be an important area for future research.

Overall, these findings raise the question of whether separate driving safety programs for male and female adolescent drivers might be advisable. For instance, results might suggest that optimal driving safety program for females vs. males might have different emphases (Choi et al., 2013), in particular in relation to distracted driving and violating the law driving. Before a decision might be made in regard to this, however, a cost-benefit analysis with key stakeholders would be critical.

One general issue important to consider when comparing the results of the present study to results of other studies in the literature is that, in global health research, research often must be conducted in different languages and hence using different linguistic versions of questionnaires (Gjersing et al., 2010). For instance, Carter et al. (2014) was conducted in the U.S. in English, Duarte and Mouro (2019) was conducted in Portugal in Portuguese, and the present study was conducted in Cambodia in Khmer. As noted above (Gjersing et al., 2010), differences in assessment instruments are necessary in order for the instruments to be valid in their context. This results in complex comparisons across studies since differences across studies could be influence by differences in the measures—as well as a myriad of cultural factors—but reflects the reality of the human species.

### Study limitations

Probably the most significant study limitation is the fact that the data are cross-sectional. It thus is not possible to determine direction of causality; i.e., whether perceptions of peers’ driving behaviors cause risky driving, or vice versa, or whether relations are due to confounding third variable effects. Thus, longitudinal research will be an important area for future research before beginning intervention development. A second study limitation is that risky driving behaviors were assessed through self-report, and the extent to which participants’ actual driving behaviors were reflected in the self-reports is unclear. Other factors (e.g., social desirability) might have influenced informants’ reports of risky driving, perceptions of peers’ driving, etc. For instance, it is possible that gender-related social desirability issues could have influenced results of our Gender analyses. One might predict that there would be higher social desirability for males than for females for aggressive driving and intoxicated driving (given male role models in many cultures, including in Cambodia) potentially leading to increased moderator effects. We did not, however, find moderator effects for Gender for aggressive driving and intoxicated driving, arguing against this hypothesis. In addition, it has generally been found that there is a strong relation between self-reports of risky driving, and police accident data and other objective indicators of driving (e.g., Ivers et al., 2009), arguing against strong social desirability effects. Nonetheless, direct assessment of social desirability levels could be a useful area for future research. Finally, data were from a single city within a single country, and their applicability to other settings is unclear. Results may, however, be useful at least as a starting point for research in similar settings within the geographic region or areas with similar cultural structures.

## Data availability statement

The data used for this study are available for study replication purposes upon reasonable request from the corresponding author, BW. Requests to access the datasets should be direct to BW, bahr.weiss@gmail.com.

## Ethics statement

This study involved human participants was approved by the Cambodian National Ethics Research Committee. The study was conducted in accordance with the local legislation and institutional requirements. Written informed consent for participation in this study was provided by the participants’ legal guardians/next of kin.

## Author contributions

BL, CL, H-MD, and BW were involved in the development of the research project design and measure selection, review of the manuscript, and analyses. BL was responsible for the project implementation, data management, initial analyses, and first draft of the manuscript. BL and CL were responsible for the project supervision. BW was responsible for the final analyses and final manuscript.

## References

[B1] American Psychiatric Association (2013). *Diagnostic and statistical manual of mental disorders*, Fifth Edn. Washington, DC: American Psychiatric Association. 10.1176/appi.books.9780890425596

[B2] Berkowtiz A. D. (2004). *The social norms approach: Theory, research and annotated bibliography.* Trumansburg, NY: Berkowtiz, A. D.

[B3] Byrne B. M. (2016). Adaptation of assessment scales in cross-national research: Issues, guidelines, and caveats. *Int. Perspect. Psychol.* 5 51–65. 10.1037/ipp0000042

[B4] Carter P. M. Bingham C. R. Zakrajsek J. S. Shope J. T. Sayer T. B. (2014). Social norms and risk perception: Predictors of distracted driving behavior among novice adolescent drivers. *J. Adolesc. Health* 54 S32–S41. 10.1016/j.jadohealth.2014.01.008 24759439 PMC7189891

[B5] Cheng A. S. K. Ng T. C. K. (2012). Risky driving and the perception of motorcycle accident causes among Chinese motorcyclists in Hong Kong. *Traffic Injury Prev.* 13 485–492. 10.1080/15389588.2012.671981 22931178

[B6] Choi M. Adams K. B. Kahana E. (2013). Self-regulatory driving behaviors: Gender and transportation support effects. *J. Women Aging* 25 104–118. 10.1080/08952841.2012.720212 23488647

[B7] Cordellieri P. Baralla F. Ferlazzo F. Sgalla R. Piccardi L. Giannini A. M. (2016). Gender effects in young road users on road safety attitudes, behaviors and risk perception. *Front. Psychol.* 7:1412. 10.3389/fpsyg.2016.01412 PMC5037216 27729877

[B8] Dahlgren P. M. (2022). Forced versus selective exposure: Threatening messages lead to anger but not dislike of political opponents. *J. Media Psychol.* 34 150–164. 10.1027/1864-1105/A000302

[B9] Duarte A. P. Mouro C. (2019). I feel safe doing it! Prevalence, risk perception, and motives for risky driving in Portugal. *Portuguese J. Public Health* 37 82–90. 10.1159/000505998

[B10] Dy K. (2016). *Driver error leading cause of road traffic accidents in Cambodia.* Washington, DC: VOA Cambodia.

[B11] Ferrer R. Klein W. M. (2015). Risk perceptions and health behavior. *Curr. Opin. Psychol.* 5 85–89. 10.1016/j.copsyc.2015.03.012 26258160 PMC4525709

[B12] Geber S. Baumann E. Czerwinski F. Klimmt C. (2021). The effects of social norms among peer groups on risk behavior: A multilevel approach to differentiate perceived and collective norms. *Commun. Res.* 48 319–345. 10.1177/0093650218824213

[B13] Gjersing L. Caplehorn J. R. Clausen T. (2010). Cross-cultural adaptation of research instruments: Language, setting, time and statistical considerations. *BMC Med. Res. Methodol.* 10:13. 10.1186/1471-2288-10-13 PMC2831007 20144247

[B14] Haghdoust Z. Masoumi G. Zavareh D. K. Ebadi A. Moslehi S. (2022). A Systematic Literature Review of Driver’s Sociocultural Factors Predisposing to Road Traffic Crashes. *Med. J. Islamic Republic Iran* 36 1–14. 10.47176/MJIRI.36.21 PMC9386747 35999932

[B15] Hambleton R. K. Merenda P. F. Spielberger C. (2005). *Adapting educational and psychological tests for cross-cultural assessment.* Mahwah: Lawrence Erlbaum Associates.

[B16] Harbeck E. L. Glendon A. I. (2018). Driver prototypes and behavioral willingness: Young driver risk perception and reported engagement in risky driving. *J. Safety Res.* 66 195–204. 10.1016/J.JSR.2018.07.009 30121106

[B17] Ivers R. Senserrick T. Boufous S. Stevenson M. Chen H. Y. Woodward M. (2009). Novice drivers’ risky driving behavior, risk perception, and crash risk: Findings from the DRIVE study. *Am. J. Public Health* 99 1638–1644. 10.2105/AJPH.2008.150367 19608953 PMC2724457

[B18] Kimmarita L. (2021). *Road toll nears 1K dead, over 2K injured this year.* Troy: Phnom Penh Post.

[B19] Latif S. A. Afgani Y. Zubair A. Som A. P. M. Wahab F. (2017). Road safety and motorcyclists’ behavior and offences by gender. *World Appl. Sci. J.* 35, 146–151. 10.5829/idosi/wasj.seiht.2017.146.151

[B20] Lucidi F. Mallia L. Giannini A. M. Sgalla R. Lazuras L. Chirico A. (2019). Riding the adolescence: Personality subtypes in young moped riders and their association with risky driving attitudes and behaviors. *Front. Psychol.* 18:300. 10.3389/fpsyg.2019.00300 PMC6387963 30833922

[B21] O’Brien F. Gormley M. (2016). Risk-perception and dangerous driving among adolescents: Outcome-and behavior-focused questions yield opposite results. *J. Adolesc.* 52 89–94. 10.1016/j.adolescence.2016.07.010 27518903 PMC5028301

[B22] Olive D. J. (2017). *Robust multivariate analysis.* Berlin: Springer Publishers, 10.1007/978-3-319-68253-2

[B23] Rhodes N. Pivik K. (2011). Age and gender differences in risky driving: The roles of positive affect and risk perception. *Accid. Anal. Prev.* 43 923–931. 10.1016/j.aap.2010.11.015 21376884

[B24] Schmidt S. A. (2012). *Youth risky driving behaviours: Advancements in measurement and theory.* Ph.D. thesis. Guelph, ON: The University of Guelph.

[B25] Simons-Morton B. G. Bingham C. R. Li K. Zhu C. Buckley L. Falk E. B. (2019). The effect of teenage passengers on simulated risky driving among teenagers: A randomized trial. *Front. Psychol.* 10:923. 10.3389/fpsyg.2019.00923 PMC6524721 31133918

[B26] Simons-Morton B. G. Raymond Bingham C. Falk E. B. Li K. Prandhan A. K. Ouimet M. C. (2014). Experimental effects of injunctive norms on simulated risky driving among teenage males. *Health Psychol.* 33 616–627. 10.1037/a0034837 24467258 PMC4189110

[B27] Song X. Yin Y. Cao H. Zhao S. Li M. Yi B. (2021). The mediating effect of driver characteristics on risky driving behaviors moderated by gender, and the classification model of driver’s driving risk. *Accid. Anal. Prev.* 153:106038. 10.1016/J.AAP.2021.106038 33631705

[B28] Tulviste T. Mizera L. De Geer B. Tryggvason M. T. (2010). Cultural, contextual, and gender differences in peer talk: A comparative study. *Scand. J. Psychol.* 51 319–325. 10.1111/J.1467-9450.2010.00809.X 20338014

[B29] United Nations Development Program – Cambodia (2021). *Road traffic accidents in Cambodia.* New York City, NY: United Nations Development Program – Cambodia.

[B30] van de Water T. Suliman S. Seedat S. (2016). Gender and cultural issues in psychiatric nosological classification systems. *CNS Spectr.* 21 334–340. 10.1017/S1092852916000122 27133577

[B31] von Beesten S. Bresges A. (2022). Effectiveness of road safety prevention in schools. *Front. Psychol.* 13:1046403. 10.3389/FPSYG.2022.1046403/FULL PMC9808381 36605279

[B32] World Health Organization (2015). *Global status report on road safety 2015.* Geneva: World Health Organization.

[B33] World Health Organization (2018). *Global status report on road safety 2018.* Geneva: World Health Organization.

[B34] Worldometer (2023). *Population of Southeast Asia.* Dover: Worldometer.

